# The Potential of Probiotics in Reducing Ventilator-Associated Pneumonia: A Literature-Based Analysis

**DOI:** 10.3390/microorganisms13040856

**Published:** 2025-04-09

**Authors:** Tao-An Chen, Ya-Ting Chuang, Szu-Chi Pai, Jin-Fu Zheng

**Affiliations:** 1 Division of Respiratory Therapy, Department of Chest Medicine, Show Chwan Memorial Hospital, Changhua 500, Taiwan; b117100045@tmu.edu.tw (T.-A.C.); deana3776@gmail.com (S.-C.P.); 2 Surgical Intensive Care Unit, Show Chwan Memorial Hospital, Changhua 500, Taiwan; s09901383@cjc.edu.tw; 3 Department of Gastroenterology Medicine, Show Chwan Memorial Hospital, Changhua 500, Taiwan

**Keywords:** ventilator-associated pneumonia, probiotics, synbiotics, intensive care unit, randomized controlled trials, systematic reviews, microbiota modulation, cost effective

## Abstract

Ventilator-associated pneumonia (VAP) remains a significant concern in intensive care units (ICUs), contributing to increased morbidity, mortality, and healthcare costs. Probiotics and synbiotics have been explored as potential preventive measures due to their ability to modulate gut microbiota, reduce pathogenic colonization, enhance immune responses, and maintain intestinal barrier integrity. While some randomized controlled trials (RCTs) suggest that specific strains, such as *Lactobacillus rhamnosus* GG and *Bifidobacterium breve*, may reduce VAP incidence, larger trials have not confirmed significant benefits. Systematic reviews and meta-analyses indicate a potential 28–38% relative risk reduction in VAP, but evidence quality remains low due to methodological limitations and study heterogeneity. Economic evaluations also question the cost effectiveness of probiotic use in ICU settings. Future research should focus on large-scale, multicenter RCTs to determine the optimal strains, dosages, and administration methods, along with standardized diagnostic criteria. Until stronger evidence emerges, probiotics should be considered an adjunctive rather than a primary VAP prevention strategy.

## 1. Introduction

Ventilator-associated pneumonia (VAP) is a type of hospital-acquired pneumonia (HAP) that develops after more than 48 h of mechanical ventilation (MV). It remains a prevalent and serious concern in intensive care units (ICUs), contributing to increased mortality risk [1]. Clinically, VAP manifests with fever, respiratory distress, increased or purulent secretions, and abnormal breath sounds. Laboratory findings often indicate worsening hypoxemia and leukocytosis, while imaging studies, such as chest X-rays or computed tomography (CT) scans, may reveal new or progressive infiltrates [2]. Diagnosis is based on the identification of new or worsening pulmonary infiltrates alongside clinical signs of infection, such as fever and increased secretions [1,2,3]. Confirmation requires pathogen identification through lower respiratory tract sampling [1].

Despite advances in critical care, VAP continues to affect approximately 10% of patients who remain on MV for more than 48 h, with incidence rates remaining stable over the past decade [4]. VAP is associated with severe complications, including prolonged hospital stays, extended ventilator dependency, and significantly higher healthcare costs—often tens of thousands of dollars more than for patients without VAP [4]. Given these substantial risks and costs, preventing VAP is of paramount importance. Currently, prevention strategies primarily involve a multi-faceted approach, with a strong emphasis on the bundle care model [5].

VAP prevention bundles consist of simple, evidence-based interventions that, when implemented collectively, enhance adherence and improve patient outcomes. Key components include strict hand hygiene compliance, daily assessments for extubation readiness in appropriate patients, maintaining endotracheal cuff pressure at a minimal occlusive level (typically 20 cmH_2_O), minimizing MV duration, reducing ICU length of stay, elevating the head of the bed to 30–45°, providing oral care with tooth brushing, and preventing condensation from reaching the patient [5].

The emphasis on bundle care stems from strong evidence supporting its effectiveness and widespread clinical recommendations. Over time, discussions have continued regarding the most effective interventions for reducing VAP incidence [6]. Among these, the potential role of probiotics and synbiotics has gained increasing attention. Research on the use of probiotics and synbiotics for VAP prevention emerged in the early 2000s [6]. Over the past two decades, numerous studies have explored their benefits; however, the findings have been inconsistent [7]. In light of these uncertainties, we conducted a comprehensive review of the literature to provide key insights and recommendations regarding the use of probiotics and synbiotics in VAP prevention.

## 2. The Impact of Probiotics on Respiratory Health and VAP

### 2.1. Probiotics, Prebiotics, and Synbiotics

In 1954, Ferdinand Vergin first introduced the term “probiotics” into medical terminology in his paper “Anti-und Probiotika” [8]. He also emphasized the adverse effects of antibiotics on intestinal flora, while beneficial bacteria have a positive effect on human health and are called “probiotics” [8]. It is well known that probiotics can promote health by stimulating the intestinal microbiota, host immunity, lower cholesterol, and several other functions, while metabolites secreted by these microorganisms (such as bacteriocins, lactic acid, and hydrogen peroxide, also known as postbiotics) can play an important role as antimicrobial agents against a variety of pathogenic bacteria [9].

**Figure 1:**
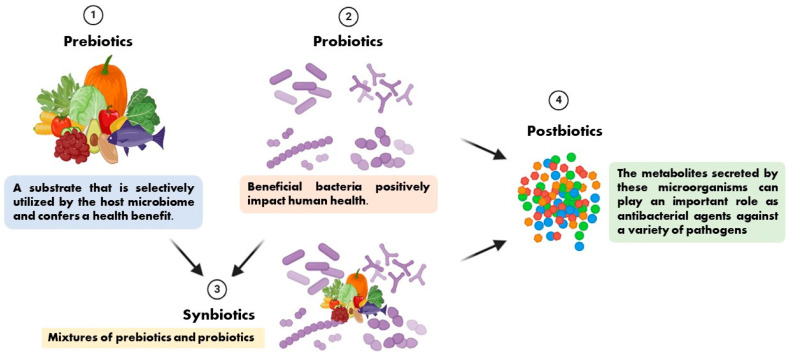
The relationships among prebiotics, probiotics, synbiotics, and postbiotics.

The term prebiotic is relatively new and was originally defined as “a substance that improves the health of the host by selectively stimulating the growth and/or activity of one or a limited number of bacteria in the colon, producing beneficial effects on the host” [10]. It has recently been defined as “a substrate that is selectively utilized by the host microbiome and confers a health benefit”. This definition expands the concept of prebiotics to potentially include non-carbohydrate substances, application to body sites other than the gastrointestinal tract, and multiple categories other than food [11]. Synbiotics are mixtures of prebiotics and probiotics used to improve human or animal health [12]. In synbiotic foods, probiotics selectively utilize prebiotics as substrates for their growth [13]. We present the relationships among prebiotics, probiotics, synbiotics, and postbiotics in Figure 1 based on the preceding description.

### 2.2. The Role of Probiotics in Respiratory Health and Ventilator-Associated Pneumonia

In summary, probiotics and synbiotics primarily reduce the incidence of VAP through the following mechanisms: (1) reducing the colonization of pathogenic bacteria in the oropharynx and stomach, thereby decreasing the risk of aspiration pneumonia; (2) stabilizing the gut microbiota and preventing bacterial translocation; (3) enhancing immune defense and modulating inflammatory responses; and (4) maintaining intestinal barrier integrity to prevent pathogen invasion into the bloodstream [14,15,16,17,18,19,20,21,22,23].

#### 2.2.1. Reduction in Pathogenic Colonization and Lowered VAP Risk

The development of VAP is closely associated with the colonization of pathogenic microorganisms in the oropharynx and stomach, with aspiration serving as a major contributor to pulmonary infection. Clinical studies have demonstrated that certain *Lactobacillus* strains, such as *Lactobacillus plantarum* 299v, can stabilize the composition of the oral microbiota, thereby reducing the growth of opportunistic pathogens in the oropharynx and subsequently decreasing the risk of aspiration into the lower respiratory tract [14,15]. Furthermore, the administration of *Lactobacillus rhamnosus* GG has been reported to significantly reduce the colonization of potentially pathogenic microorganisms in the stomach and oropharynx among critically ill patients [16,17], which may play a role in reducing the risk of VAP caused by multidrug-resistant Gram-negative bacteria, such as *Pseudomonas aeruginosa* and *Acinetobacter baumannii* [18]. In addition, oral supplementation with *Lactobacillus casei rhamnosus* strain 35 has been found to delay *P. aeruginosa* colonization in the respiratory tract, providing an alternative strategy for preventing colonization by this pathogen, particularly in long-term MV patients [19]. The use of synbiotic formulations containing *Bifidobacterium breve* and *Lactobacillus casei*, such as Yakult BL Seichoyaku (Yakult Honsha, Tokyo, Japan), has also been shown to enhance the production of short-chain fatty acids (SCFAs), including acetate, which further inhibits the overgrowth of enteric pathogens in the gastrointestinal tract [20].

#### 2.2.2. Stabilization of Gut Microbiota and Prevention of Bacterial Translocation

Critically ill patients are prone to gut microbiota dysbiosis, which promotes the overgrowth of opportunistic pathogens, including members of the *Enterobacteriaceae* family. This microbial imbalance increases the likelihood of bacterial translocation across the intestinal barrier, subsequently contributing to systemic infections and the development of VAP [21,22]. The administration of synbiotic formulations containing multiple *Lactobacillus* and *Bifidobacterium* species, such as Synbiotic 2000 FORTE (Medipharm, Kagerod, Sweden and Des Moines, IA, USA), has been demonstrated to promote the growth of commensal gut bacteria while suppressing the proliferation of opportunistic pathogens, thereby reducing the risk of bacterial translocation [21,22]. Additionally, the administration of a commercially available synbiotic formulation, FamiLact 2plus (Zist Takhmir Company, Tehran, Iran), has been reported to modulate gut microbiota composition, mitigate antibiotic-associated dysbiosis, and consequently decrease the incidence of VAP [23].

#### 2.2.3. Enhancement of Immune Function and Modulation of Inflammatory Responses

Probiotics exert immunomodulatory effects by competitively inhibiting pathogen colonization and interacting with host immune signaling pathways. One proposed mechanism involves the interaction between microbial-associated molecular patterns (MAMPs) from probiotic species and host pattern recognition receptors (PRRs), including Toll-like receptors (TLRs), leading to the activation of innate immune defenses [20,22]. Experimental studies have shown that *Lactobacillus casei* administration enhances interleukin-12 (IL-12) production in the intestinal mucosa, thereby augmenting natural killer (NK) cell activity [18,20]. Additionally, preclinical investigations have demonstrated that *Bifidobacterium breve* modulates pulmonary inflammation through acetate production, attenuating oxidative stress and cellular damage in the lung tissue [18]. Moreover, *Lactobacillus plantarum* has been shown to metabolize arginine into nitric oxide (NO), which facilitates mucus secretion and regulates gastrointestinal motility, contributing to the maintenance of intestinal barrier integrity [18].

#### 2.2.4. Maintenance of Intestinal Barrier Integrity and Prevention of Pathogen Translocation

Intestinal barrier dysfunction is a hallmark of critical illness and is often associated with the translocation of enteric pathogens and endotoxins, such as lipopolysaccharide (LPS), into the bloodstream. This process can trigger systemic inflammatory response syndrome (SIRS), thereby increasing the risk of VAP [22]. Studies have demonstrated that the administration of specific probiotic strains, including *Bifidobacterium breve* and *Lactobacillus casei*, enhances the expression of tight junction proteins such as claudin-1, occludin, and zonula occludens-1 (ZO-1), thereby reinforcing the integrity of the intestinal epithelial barrier and reducing microbial translocation [20,22]. These protective effects have been particularly observed in patients with traumatic brain injury (TBI), where early probiotic administration mitigates dysbiosis-induced pathogen overgrowth [18].

## 3. Efficacy of Probiotics and Synbiotics in VAP Prevention: Clinical Insights

This systematic review was conducted following the PRISMA (Preferred Reporting Items for Systematic Reviews and Meta-Analyses) guidelines to ensure a structured and transparent methodology. The primary research question was “In critically ill patients, can probiotics or synbiotics reduce the incidence of VAP compared to standard care”?

The bibliographic search was performed using PubMed, covering studies published up to 25 January 2025. The search strategy included the terms (“probiotic s” [All Fields] OR “probiotical” [All Fields] OR “probiotics” [MeSH Terms] OR “probiotics” [All Fields] OR “probiotic” [All Fields] OR (“synbiotics” [MeSH Terms] OR “synbiotics” [All Fields] OR “synbiotic” [All Fields])) AND (“pneumonia, ventilator associated” [MeSH Terms] OR (“pneumonia” [All Fields] AND “ventilator associated” [All Fields]) OR “ventilator-associated pneumonia” [All Fields] OR (“ventilator” [All Fields] AND “associated” [All Fields] AND “pneumonia” [All Fields]) OR “ventilator associated pneumonia” [All Fields]). The search was limited to articles published in English.

Two independent reviewers (T.-A.C. and Y.-T.C.) conducted the search and study selection. Any discrepancies were resolved by consulting a third reviewer (J.-F.Z.). The inclusion criteria were (1) randomized controlled trials (RCTs) and cohort studies assessing the use of probiotics or synbiotics for VAP prevention in critically ill adult patients and (2) studies reporting incidence rates of VAP as an outcome. The exclusion criteria included (1) studies conducted in neonatal or pediatric populations; (2) review articles, meta-analyses, or study protocols; (3) studies that did not report VAP incidence as an outcome; (4) not full text; (5) same research project with similar primary outcomes.

**Figure 2:**
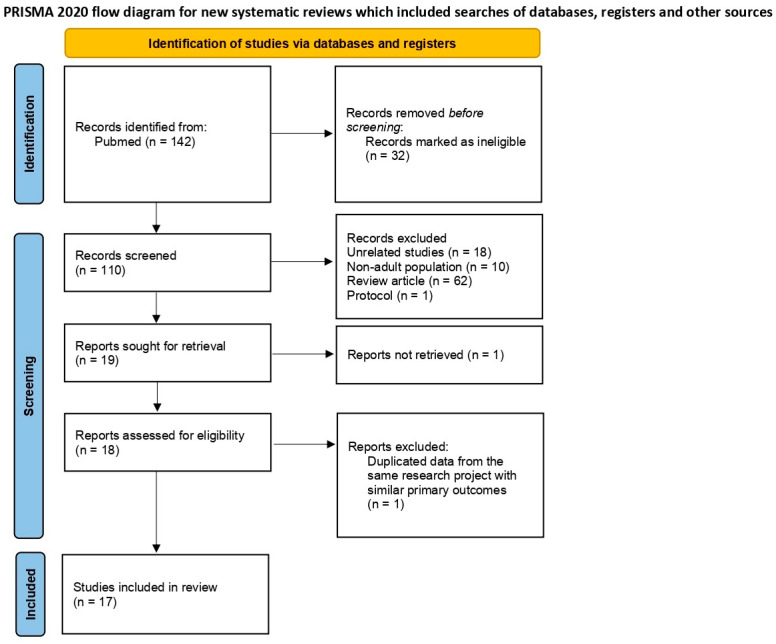
PRISMA 2020 flow diagram: study selection and inclusion process for a systematic review.

A total of 142 articles were identified in the initial PubMed search. After removing 32 ineligible records, 110 articles were screened based on titles and abstracts. Of these, 91 were excluded for reasons such as being unrelated (*n* = 18), focusing on non-adult populations (*n* = 10), being review articles (*n* = 62), or study protocols (*n* = 1). Nineteen articles were assessed for full-text retrieval, but one was not retrieved. After full-text assessment, one study was excluded due to duplicate data from the same research project with similar primary outcomes, leaving seventeen studies included in the final review. Figure 2 presents the PRISMA flow diagram summarizing the study selection process.

We summarized studies that evaluated the effectiveness of probiotics and synbiotics in preventing VAP among ICU patients, focusing on study design, patient characteristics, dosage, and administration methods. While some trials demonstrated a significant reduction in VAP incidence, others reported no clear benefit, highlighting the variability in outcomes across different probiotic strains and synbiotic formulations [16,20,23]. Differences in sample size, inclusion criteria, and control conditions may have influenced these results, as larger studies with 150 and 2650 patients often failed to replicate the positive findings observed in smaller trials [17,24,25]. The mode of administration also varied, including nasogastric or enteral feeding, oropharyngeal slurry, and oral care, which may impact colonization effectiveness and overall VAP prevention [14,15,16]. Additionally, some studies reported a delayed onset of VAP or reduced pathogen colonization, particularly for *Pseudomonas aeruginosa* and *Acinetobacter baumannii*, but these effects were not consistently observed across all trials [18,19,21]. Given these inconsistencies, further research is required to establish standardized protocols for strain selection, administration routes, and dosing regimens to optimize the clinical utility of probiotics and synbiotics in VAP prevention.

### 3.1. Influence of Study Design and Patient Characteristics on Probiotic and Synbiotic Effectiveness

Multiple randomized controlled trials (RCTs) have assessed the efficacy of probiotics and synbiotics in preventing VAP among ICU patients, but the results remain inconsistent. A study involving 208 ICU patients receiving *Lactobacillus casei rhamnosus* demonstrated delayed acquisition of *Pseudomonas aeruginosa*-associated VAP compared to placebo, suggesting a potential protective effect [19]. Similarly, another study with 138 ICU patients found that *Lactobacillus rhamnosus* GG significantly reduced VAP incidence when administered via both oropharyngeal slurry and nasogastric tube [16]. However, larger trials, including studies with 150 and 2650 patients, failed to replicate this effect, indicating that differences in study design, patient populations, and administration protocols may influence outcomes [17,24,25]. Moreover, the use of *Lactobacillus plantarum* 299 for oral care in trials with 44 and 137 patients reduced bacterial colonization but did not significantly impact VAP incidence [14,15]. These findings suggest that while probiotics may influence airway microbiota composition, their direct role in preventing VAP remains uncertain.

The efficacy of synbiotics has also been explored in ICU patients, with varying results. A trial administering Synbiotic 2000 FORTE, a formulation containing *Pediococcus pentosaceus*, *Leuconostoc mesenteroides*, *Lactobacillus paracasei*, and *Lactobacillus plantarum*, to 72 and 259 patients found no significant reduction in VAP incidence compared to placebo [21,26]. In contrast, studies evaluating alternative synbiotic formulations demonstrated more promising results. A trial with 72 ICU patients diagnosed with sepsis reported a significant reduction in VAP incidence following the administration of *Bifidobacterium breve* and *Lactobacillus casei* via nasal tube feeding [20]. Additionally, a study involving 80 ICU patients receiving synbiotics, FamiLact 2plus, which contains multiple *Lactobacillus* and *Bifidobacterium* strains, showed a significant reduction in VAP incidence [23]. Another trial with 235 patients using *Bacillus subtilis* and *Enterococcus faecalis* also reported a significant reduction in VAP incidence, suggesting that strain selection may play a critical role in determining clinical efficacy [27]. However, a trial involving 100 ICU patients receiving a combination of *Lactobacillus* and *Bifidobacterium* species demonstrated a significant reduction in VAP incidence but no difference in timing, indicating that additional factors, such as immune modulation and microbiome interactions, may influence outcomes [28]. We have compiled and summarized the studies in Table 1.

### 3.2. Impact of Dosage and Administration Strategies on VAP Outcomes

The mode of administration and dosage of probiotics and synbiotics may be critical factors influencing their effectiveness in preventing VAP. Studies evaluating *Lactobacillus casei rhamnosus* and *Lactobacillus rhamnosus* GG have used nasogastric tube feeding at doses ranging from 10^9^ to 10^10^ colony-forming units (CFUs) per administration, with one study reporting delayed *Pseudomonas aeruginosa*-associated VAP acquisition and another showing a significant reduction in overall VAP incidence [16,19]. However, similar administration methods in larger studies with 150 and 2650 patients did not demonstrate a significant effect, suggesting that factors beyond dosage and delivery route, such as patient heterogeneity and ICU conditions, may influence probiotic efficacy [17,24,25]. Additionally, oral care administration using gauze swabs soaked with *Lactobacillus plantarum* 299 at 10^10^ CFUs twice daily resulted in reduced colonization rates but did not significantly prevent VAP development [14,15]. The inconsistency in the results suggests that probiotics may be more effective when administered directly to the gastrointestinal tract rather than solely targeting the oral cavity.

Synbiotic administration methods and dosages also varied across studies, potentially contributing to the observed differences in efficacy. Studies evaluating Synbiotic 2000 FORTE administered the formulation via nasogastric or orogastric tube feeding at 10^10^ bacteria per sachet twice daily, but no significant reduction in VAP incidence was observed [21,26]. In contrast, the administration of *Bifidobacterium breve* and *Lactobacillus casei* at 2 × 10^8^ CFUs/g, 3 g/day via nasal tube feeding resulted in a significant reduction in VAP incidence and its cumulative rate [20]. A similar reduction in VAP incidence was observed in a study utilizing synbiotics, FamiLact 2plus, administered via enteral feeding at 10^9^ CFUs twice daily [23]. However, despite these positive findings, another trial using an alternative synbiotic formulation at 2 × 10^10^ CFUs per day found no significant reduction in VAP incidence [29]. These findings indicate that synbiotic efficacy may be influenced by strain composition, administration route, and host-specific factors, underscoring the need for further large-scale, standardized trials to determine the optimal conditions for their use in VAP prevention. We have compiled and summarized the studies in Table 2.

## 4. Weighing the Evidence: Systematic Reviews and Meta-Analysis on Probiotics and Synbiotics for VAP Reduction

There are many studies supporting the use of probiotics and synbiotics for VAP prevention; however, existing studies demonstrate considerable variability in outcomes [14,16,18,19,20,22,23,27,28]. Differences in probiotic strains, dosages, and administration methods contribute to inconsistencies, making it challenging to establish definitive clinical guidelines. While multi-strain formulations and synbiotics may enhance efficacy through synergistic effects, their specific advantages over single-strain probiotics remain uncertain [20,21,22,23,26,29]. Additionally, concerns about study quality, patient heterogeneity, and methodological limitations highlight the need for more rigorous trials [6,18,28].

Despite these challenges, conducting systematic reviews and meta-analyses is essential to synthesize existing evidence, identify patterns across diverse studies, and assess the overall efficacy of probiotics and synbiotics in VAP prevention. By integrating data from multiple trials, these analyses can help mitigate inconsistencies, refine clinical recommendations, and guide future research directions. Moreover, they provide a structured framework to evaluate study quality, address potential biases, and enhance the reliability of conclusions, ultimately supporting evidence-based decision making in critical care settings.

### 4.1. Effectiveness of Probiotics and Synbiotics in Reducing VAP Incidence

Several systematic reviews and meta-analyses have evaluated the role of probiotics in preventing VAP in critically ill patients. An early meta-analysis included five RCTs with a total of 689 patients, reporting that probiotics significantly reduced VAP incidence (OR 0.55–0.61) compared to controls [6]. A later review analyzed eight RCTs involving 1083 patients and also found a protective effect of probiotics (OR 0.70, 95% CI 0.52–0.95) [30]. These findings suggest that probiotics may be beneficial in reducing VAP incidence, although study limitations and methodological variations must be considered.

A more recent meta-analysis included 14 RCTs with 1975 patients and demonstrated a significant reduction in VAP incidence (OR 0.62, 95% CI 0.45–0.85) [31]. However, when restricting the analysis to double-blinded studies, the effect was no longer statistically significant (OR 0.72, 95% CI 0.44–1.19), highlighting potential biases in previous studies [31]. Similarly, another review of 18 RCTs (4893 patients) found a 32% relative risk reduction in VAP (RR 0.68, 95% CI 0.55–0.84) but noted that the overall quality of evidence remained low due to study heterogeneity and potential bias [7]. We have compiled and summarized the studies in Table 3.

### 4.2. Limitations and Heterogeneity in Existing Studies

The effectiveness of probiotics in VAP prevention may be influenced by the use of multi-strain formulations versus single-strain probiotics. Synbiotics, which combine probiotics with prebiotics, such as Synbiotic 2000 FORTE, have been reported to yield greater reductions in VAP incidence compared to single-strain probiotics [30]. This suggests that the presence of multiple bacterial species, along with prebiotic components, may enhance colonization resistance against pathogenic bacteria in the respiratory tract. However, some meta-analyses indicate that while multi-strain formulations appear more effective in reducing overall infection rates, their specific impact on VAP prevention remains inconsistent [7].

The choice of probiotic strains and formulations significantly impacts the observed effects on VAP prevention. *Lactobacillus* and *Bifidobacterium* species are the most commonly studied probiotics, with *Lactobacillus rhamnosus* GG and *Lactobacillus plantarum* demonstrating promising results in reducing VAP incidence [7]. However, the effectiveness of different strains varies, and certain formulations may be more beneficial than others. The inclusion of *Saccharomyces boulardii* in some studies raises questions about whether yeast-based probiotics offer additional advantages over bacterial probiotics in critically ill patients [33].

The variability in study designs, patient populations, probiotic strains, dosages, and administration routes contributes to inconsistent findings across meta-analyses. For example, one study found that the exclusion of a study applying probiotics only to the oral cavity did not alter the beneficial effect on VAP incidence (OR 0.62) [6]. In contrast, another review noted that trials using Synbiotic 2000 FORTE consistently demonstrated greater efficacy in reducing VAP (OR 0.44, 95% CI 0.24–0.79) [30]. This suggests that the effectiveness of probiotics may depend on specific formulations and delivery methods.

The most comprehensive meta-analysis included 65 RCTs (17 related to VAP), encompassing 8483 patients. The findings showed a 6.9% absolute reduction in VAP incidence (RR 0.72, 95% CI 0.59–0.89) but also highlighted significant heterogeneity in the included trials [33]. The lack of standardized diagnostic criteria for VAP and variations in patient severity further complicate the interpretation of results. Notably, sensitivity analyses excluding high risk-of-bias studies found no significant effect of probiotics, reducing confidence in the pooled estimate [33]. We have compiled and summarized the studies in Table 4.

### 4.3. Clinical and Economic Considerations

Beyond VAP prevention, probiotics may offer economic benefits by reducing antibiotic use and healthcare costs. A health economic evaluation estimated a cost–benefit ratio with a willingness-to-pay threshold of USD 50,000–100,000 per VAP case averted, with a median cost of USD 15,958 per case (range: USD 7000–35,000) [32]. However, this study relied on limited data and was not a direct meta-analysis of VAP prevention. Moreover, another study reported that probiotic administration led to a modest reduction in antibiotic use duration (mean difference: −1.44 days, 95% CI [−2.88, −0.01], *p* = 0.05), indicating potential ancillary benefits [31].

However, subsequent large-scale studies have raised concerns about the cost effectiveness of probiotics in ICU settings. In the E-PROSPECT study, probiotics were not identified as a major cost driver, whereas ICU hoteling, ICU nursing, ward nursing, ward hoteling, and other personnel costs played a more significant role. Notably, further cost effectiveness analyses concluded that probiotics were not cost effective in ICU settings, as their impact on reducing overall healthcare expenditures was minimal despite potential clinical benefits [25].

### 4.4. Conclusion and Future Directions for Systematic Reviews and Meta-Analyses

While probiotics show promise in reducing VAP incidence, the certainty of evidence remains low due to methodological limitations, heterogeneity, and potential biases in existing studies. Current meta-analyses suggest a potential 28–38% relative risk reduction in VAP with probiotic use, but results vary depending on study design and patient population [6,7,30,31,32,33]. Although a large-scale study involving more than 2000 participants was conducted, and subsequent systematic reviews and meta-analyses incorporated its findings, the number of newly published RCTs since its publication has remained limited up until our review of this topic [18,23,24]. Consequently, their impact on recent systematic reviews and meta-analyses has likely been minimal. Further large-scale, high-quality RCTs are needed to determine the optimal probiotic strains, dosages, and duration of administration for effective VAP prevention. Additionally, future research should include standardized diagnostic criteria and explore the safety of probiotics in immunocompromised patients, as this population was largely excluded from previous studies [7,33].

Furthermore, while research on probiotics and synbiotics continues to expand, evidence regarding the role of postbiotics in VAP prevention remains insufficient. Future systematic reviews and meta-analyses should incorporate studies on postbiotics to evaluate their potential efficacy and clinical applicability.

## 5. Future Directions in Probiotics and Synbiotics for VAP Prevention

The use of probiotics and synbiotics for preventing VAP shows promise but faces several challenges. Many studies are single-center trials, leading to biases related to local clinical practices and patient populations, which limit the generalizability of the findings [16,19,26]. Additionally, variations in VAP diagnostic criteria across studies result in inconsistent reported incidence rates, complicating comparisons [27,31]. Due to the lack of standardized definitions, reported VAP incidence varies significantly, ranging from 4% to 42%, depending on the criteria used [27].

Another challenge is the heterogeneity in probiotic strains, dosages, and administration routes used in different trials [7,16,27,31]. Furthermore, probiotic therapy requires adherence and is inherently susceptible to human error [16]. Many studies have low methodological quality, with issues related to randomization, allocation concealment, and blinding, which may affect the reliability of results [7,30,31]. Additionally, the small sample sizes of many trials limit statistical power [14,16,20,21,23,27].

Systematic reviews and meta-analyses highlight the heterogeneity in reported data, making it difficult to draw definitive conclusions [6,7,30,31,32,33]. Funding bias from manufacturer-sponsored studies and the lack of patient-level data further reduce the credibility of the evidence [32]. Moreover, no studies have assessed the impact of probiotics on immunocompromised patients, leaving an important population unexamined [7].

Future research should prioritize large-scale, multicenter RCTs to address these gaps [19,28,30,31]. It is also essential to consider the influence of geographic, racial, and lifestyle factors on gut microbiota composition, as these may affect probiotic efficacy [20,34]. Standardizing VAP diagnostic criteria and ensuring adequate power to assess clinical outcomes will be crucial [27,31]. Additionally, economic evaluations should be conducted to determine the cost effectiveness of probiotics in VAP prevention [25,32]. Some guidelines advise against prescribing probiotics to specific populations, while others neither recommend nor oppose their routine use as part of standard VAP prevention strategies [35,36,37]. Addressing these challenges through rigorous study designs and standardized methodologies will help clarify the role of probiotics and synbiotics in VAP prevention.

## 6. Conclusions

Based on this comprehensive analysis, probiotics and synbiotics show the potential to reduce the incidence of VAP, but the current research results remain highly variable. While some randomized RCTs suggest that specific strains (such as *Lactobacillus rhamnosus* GG and *Bifidobacterium breve*) may effectively reduce VAP incidence, larger trials have failed to confirm significant preventive effects. This inconsistency may be due to differences in study design, patient characteristics, dosage, administration routes, and ICU environments.

Additionally, literature reviews and systematic reviews indicate that the preventive effects of probiotics and synbiotics may be related to their ability to modulate gut microbiota, reduce pathogenic colonization, enhance immune function, and maintain intestinal barrier integrity. However, factors such as strain selection, dosage, administration method (oral, enteral feeding, or topical application), baseline patient conditions, and concurrent preventive measures (such as oral care and antibiotic use) may influence their effectiveness.

Although systematic reviews and meta-analyses suggest that probiotics may reduce VAP incidence by approximately 28–38% in relative risk reduction, the overall quality of evidence remains low due to methodological limitations and high heterogeneity among studies. Economic evaluations also indicate that the use of probiotics may not significantly reduce overall ICU healthcare costs. Therefore, probiotics should not yet be considered a standard VAP prevention strategy but rather an adjunctive measure.

Future research should prioritize large-scale, multicenter RCTs to identify the optimal probiotic and synbiotic strains, dosages, and administration timing while also standardizing VAP diagnostic criteria to minimize study heterogeneity. Additionally, the safety of probiotics in immunocompromised patients remains underexplored and should be a key focus of future investigations. While research on probiotics and synbiotics continues to grow, evidence on the role of postbiotics in VAP prevention remains limited. Therefore, future research should also consider postbiotics as a noteworthy area of study, evaluating their potential efficacy and clinical applicability.

## Figures and Tables

**Table 1:** Summary of clinical trials investigating probiotics and synbiotics for VAP prevention.

Study (Year)	Study Design	Sample Size	Patient Population	Probiotic Strain(s)/Synbiotics	Control Group	Reference
Forestier et al. (2008)	RCT ^¶^ (prospective, randomized, double-blind, placebo-controlled pilot study)	208	ICU ^‡^ >18 years old and hospital stay > 48 h	Probiotics: *Lactobacillus * *casei rhamnosus*	Placebo	[19]
Klarin et al. (2008)	RCT ^¶^ (randomized controlled open pilot study)	44	ICU ^‡^ >18 years old and MV ^§^ time > 24 h	Probiotics: *Lactobacillus * *plantarum* 299 (LP299)	CHX ^†^ solution oral care	[14]
Knight et al. (2009)	RCT ^¶^ (prospective, randomized, double-blind, placebo-controlled trial)	259	ICU ^‡^ >16 years old and MV ^§^ time > 48 h	Synbiotic 2000 FORTE (*Pediococcus pentosaceus*, *Leuconostoc mesenteroides*, *Lactobacillus paracasei* subsp. *paracasei * *Lactobacillus plantarum*)	Placebo	[26]
Giamarellos- Bourboulis et al. (2009)	RCT ^¶^ (randomized, Double-blind, placebo-controlled, multicenter clinical trial)	72	ICU ^‡^ Severe multiple injuries with MV ^§^	Synbiotic 2000 FORTE (*Pediococcus pentosaceus*, *Leuconostoc mesenteroides*, *Lactobacillus paracasei* subsp. *paracasei * *Lactobacillus plantarum*)	Placebo	[21]
Barraud et al. (2010)	RCT ^¶^ (randomized, Double-blind, placebo-controlled trial)	167	ICU ^‡^ >18 years old and MV ^§^ time > 48 h	Synbiotics: Ergyphilus (*Lactobacillus rhamnosus* GG, *Lactobacillus casei*, *Lactobacillus acidophilus*, *Bifidobacterium bifidum*)	Placebo	[29]
Morrow et al. (2010)	RCT ^¶^ (prospective, randomized, double-blind, placebo-controlled trial)	138	ICU ^‡^ >19 years old and MV ^§^ time > 72 h (tracheostomy excluded)	Probiotics: *Lactobacillus rhamnosus* GG	Placebo	[16]
Zeng et al. (2016)	RCT ^¶^ (prospective, randomized, open-label, controlled multicenter study)	235	ICU ^‡^ >18 years old and MV ^§^ time > 48 h	Probiotics: *Bacillus subtilis* *Enterococcus faecalis*	Only standard strategies	[27]
Cook et al. (2016)	RCT ^¶^ (randomized concealed blinded parallel trial)	150	ICU ^‡^ >18 years old and MV ^§^ time > 72 h	Probiotics: *Lactobacillus rhamnosus* GG	Placebo	[17]
Shimizu et al. (2018)	Cohort study (retrospective observational study)	179	ICU ^‡^ MV	Synbiotics: *Bifidobacterium breve*, *Lactobacillus casei*,	Synbiotics not used	[22]
Shimizu et al. (2018)	RCT ^¶^ (randomized, Single-blind study)	72	ICU ^‡^ Sepsis >16 years old and MV ^§^	Synbiotics: *Bifidobacterium breve*, *Lactobacillus casei*,	Synbiotics not used	[20]
Klarin et al. (2018)	RCT ^¶^ (prospective, randomized, multicenter, controlled open trial)	137	ICU ^‡^ >18 years old and MV ^§^ time > 24 h (tracheostomy excluded)	Probiotics: *Lactobacillus * *plantarum* 299 (LP299)	CHX ^†^ solution oral care	[15]
Mahmoodpoor et al. (2019)	RCT ^¶^ (prospective, randomized, double-blind, placebo-controlled trial)	100	ICU ^‡^ >18 years old and MV ^§^ time > 48 h (tracheostomy excluded)	Probiotics: *Lactobacillus* species (*casei*, *acidophilus*, *rhamnosus*, *bulgaricus*), *Bifidobacterium* species (*breve*, *longum*), *Streptococcus thermophilus*	Placebo	[28]
Johnstone et al. (2021)	RCT ^¶^ (prospective, randomized, blind, placebo-controlled trial)	2650	ICU ^‡^ >18 years old and MV ^§^ time > 72 h	Probiotics: *Lactobacillus rhamnosus* GG	Placebo	[24]
Tsilika et al. (2022)	RCT ^¶^ (randomized, double-blind, multicenter, placebo-controlled trial)	112	ICU ^‡^ Multi-trauma patients requiring MV ^§^ 18–80 years old	Probiotics: *Lactobacillus acidophilus* LA-5, *Lactobacillus plantarum* UBLP-40, *Bifidobacterium animalis* subsp. *lactis* BB-12, *Saccharomyces boulardii* Unique-28	Placebo	[18]
Lau et al. (2022)	RCT ^¶^ (prospective, randomized, blind, placebo-controlled trial)	2650	ICU ^‡^ >18 years old and MV ^§^ time > 72 h	Probiotics: *Lactobacillus rhamnosus* GG	Placebo	[25]
Kasiri et al. (2023)	RCT ^¶^ (randomized, triple-blind, single-center, placebo-controlled trial)	80	ICU ^‡^ MV ^§^ time > 48 h	Synbiotics: FamiLact 2plus: *Lactobacillus acidophilus*, *Lactobacillus casei*, *Lactobacillus rhamnosus*, *Lactobacillus salivarius*, *Lactobacillus reuteri*, *Bifidobacterium lactis*, *Bifidobacterium longum*, *Bifidobacterium bifidum*	Placebo	[23]

^†^ CHX: chlorhexidine; ^‡^ ICU: intensive care unit; ^§^ MV: mechanical ventilation; ^¶^ RCT: randomized controlled trial.

**Table 2:** Administration methods, dosages, and outcomes of probiotic and synbiotic interventions for VAP prevention.

Study (Year)	Probiotic Strain(s)/Synbiotics	Administration Route and Dose	VAP			Reference
			Outcomes Mention (Primary/ Secondary)	Conclusion on Effect	Assessment of Prevention (Positive/ Negative)	
Forestier et al. (2008)	Probiotics: *Lactobacillus * *casei rhamnosus*	Nasogastric tube feeding (10^9^ CFUs ^†^) twice daily	Primary	Delayed *P. aeruginosa* VAP ^‡^ acquisition	Positive	[19]
Klarin et al. (2008)	Probiotics: *Lactobacillus * *plantarum* 299 (LP299)	Gauze swabs oral care (10^10^ CFUs ^†^) twice daily	Secondary	Reduced colonization	Positive	[14]
Knight et al. (2009)	Synbiotic 2000 FORTE (*Pediococcus pentosaceus*, *Leuconostoc mesenteroides*, *Lactobacillus paracasei* subsp. *paracasei * *Lactobacillus plantarum*)	Nasogastric/ orogastric tube feeding (10^10^ bacteria/sachet) twice daily	Primary	Not significantly reduced VAP ^‡^ incidence	Negative	[26]
Giamarellos- Bourboulis et al. (2009)	Synbiotic 2000 FORTE (*Pediococcus pentosaceus*, *Leuconostoc mesenteroides*, *Lactobacillus paracasei* subsp. *paracasei * *Lactobacillus plantarum*)	Nasogastric tube/ gastrostomy feeding (10^11^ CFUs ^†^) once daily	Secondary	Not significantly reduced VAP ^‡^ incidence (but may reduce *A. baumannii*-induced VAP ^‡^)	Negative	[21]
Barraud et al. (2010)	Synbiotics: Ergyphilus (*Lactobacillus rhamnosus* GG, *Lactobacillus casei*, *Lactobacillus acidophilus*, *Bifidobacterium bifidum*)	Enteral feeding tube (2 × 10^10^ revivable bacteria) once daily	Secondary	Not directly mentioned (but the incidence of VAP ^‡^ showed no significance)	Negative	[29]
Morrow et al. (2010)	Probiotics: *Lactobacillus rhamnosus* GG	One as a slurry to the oropharynx and the other via nasogastric tube feeding (2 × 2 × 10^9^ CFUs ^†^) twice daily	Primary	Significantly reduced VAP ^‡^ incidence	Positive	[16]
Zeng et al. (2016)	Probiotics: *Bacillus subtilis* *Enterococcus faecalis*	Nasogastric tube feeding (5 × 10^9^ CFUs ^†^) three times daily	Primary	Significantly reduced and delayed the onset of VAP ^‡^	Positive	[27]
Cook et al. (2016)	Probiotics: *Lactobacillus rhamnosus* GG	Gastric/ duodenal tube feeding (10^10^ CFU ^†^) twice daily	Primary	No clear conclusion on VAP ^‡^ prevention	Not applicable	[17]
Shimizu et al. (2018)	Synbiotics: *Bifidobacterium breve*, *Lactobacillus casei*,	Nasal tube feeding (2 × 10^8^ living bacteria/g × 3 g/day) within 3 days	Primary	Trend of reduced VAP ^‡^, but not significant	Positive	[22]
Shimizu et al. (2018)	Synbiotics: *Bifidobacterium breve*, *Lactobacillus casei*,	Nasal tube feeding (2 × 10^8^ living bacteria/g × 3 g/day) within 3 days	Primary	Significantly reduced VAP ^‡^ incidence and its cumulative rate	Positive	[20]
Klarin et al. (2018)	Probiotics: *Lactobacillus * *plantarum* 299	Gauze swabs oral care (10^10^ CFUs ^†^) twice daily	Secondary	Not significantly reduced VAP ^‡^ incidence	Negative	[15]
Mahmoodpoor et al. (2019)	Probiotics: *Lactobacillus* species (*casei*, *acidophilus*, *rhamnosus*, *bulgaricus*), *Bifidobacterium* species (*breve*, *longum*), *Streptococcus thermophilus*	Feeding tube feeding (10^10^ bacteria consisting) twice daily	Primary	Significantly reduced VAP ^‡^ incidence, but no timing difference	Positive	[28]
Johnstone et al. (2021)	Probiotics: *Lactobacillus rhamnosus* GG	Enteral (10^10^ CFUs ^†^) twice daily	Primary	No significant reduction in VAP ^‡^ incidence	Negative	[24]
Tsilika et al. (2022)	Probiotics: *Lactobacillus acidophilus* LA-5, *Lactobacillus plantarum* UBLP-40, *Bifidobacterium animalis* subsp. *lactis* BB-12, *Saccharomyces boulardii* Unique-28	One as a slurry to the oropharynx and the other via nasogastric or gastrostomy tube feeding (2 × 5.5 × 10^9^ CFUs ^†^) twice daily	Primary	Significantly reduced VAP ^‡^ incidence (reducing the risk of *P. aeruginosa* and *A. baumannii*-induced VAP ^‡^)	Positive	[18]
Lau et al. (2022)	Probiotics: *Lactobacillus rhamnosus* GG	Enteral (10^10^ CFUs ^†^) twice daily	Primary	No significant reduction in VAP ^‡^ incidence costly, not cost effective	Negative	[25]
Kasiri et al. (2023)	Synbiotics: FamiLact 2plus: *Lactobacillus acidophilus*, *Lactobacillus casei*, *Lactobacillus rhamnosus*, *Lactobacillus salivarius*, *Lactobacillus reuteri*, *Bifidobacterium lactis*, *Bifidobacterium longum*, *Bifidobacterium bifidum*	Intestinal feeding (10^9^ CFUs ^†^) twice daily	Primary	Significantly reduced VAP ^‡^ incidence	Positive	[23]

^†^ CFU: colony-forming unit; ^‡^ VAP: ventilator-associated pneumonia.

**Table 3:** Probiotics and synbiotics for VAP risk: systematic reviews and meta-analyses.

Study (Year)	Study Type	No. of RCTs	Sample Size	Primary Outcomes (VAP Incidence)	Conclusion and Recommendation	Reference
Siempos et al. (2010)	Systematic Review and Meta-analysis	5	689	1. Fixed effect model: OR ^‡^ 0.61 (95% CI ^†^ 0.41–0.91) 2. Random effects model: OR ^‡^ 0.55 (95% CI ^†^ 0.31–0.98)	Probiotics reduce VAP ^※^ risk. Prior reviews were inconclusive due to limited data.	[6]
Bo et al. (2014)	Systematic Review and Meta-analysis	8	1083	OR ^‡^ 0.70 (95% CI ^†^ 0.52–0.95)	Probiotics may lower VAP ^※^ risk but should be used cautiously. Further research is needed.	[30]
Lau et al. (2020)	Systematic Review	7 Studies (1 study mentions VAP)	Not specified	Cost–benefit analysis	Probiotics reduce VAP ^※^ incidence, but the economic impact varies.	[32]
Su et al. (2020)	Systematic Review and Meta-analysis	14	1975	1. Total VAP incidence: OR ^‡^ 0.62 (95% CI ^†^ 0.45–0.85) 2. Not significant in double-blind studies: OR ^‡^ 0.72 (95% CI ^†^ 0.44–1.19)	Probiotics significantly lower VAP ^※^ but need verification in large trials.	[31]
Cheema et al. (2022)	Systematic Review and Meta-analysis	18	4893	RR ^⁂^ 0.68 (95% CI ^†^ 0.55–0.84)	Probiotics may reduce VAP ^※^ incidence, but evidence quality is low.	[7]
Sharif et al. (2022)	Systematic Review and Meta-analysis	65 (17 RCTs ^§^ mention VAP ^※^)	8483 (2367 patients included)	RR ^⁂^ 0.72 (95% CI ^†^ 0.59–0.89) RD ^¶^ 6.9% reduction (95% CI ^†^ 2.7–10.2%)	Probiotics appear effective, but evidence certainty is low.	[33]

^†^ CI: confidence interval; ^‡^ OR: odds ratio; ^§^ RCTs: randomized controlled trials; ^¶^ RD: risk difference; ⁂ RR: risk ratio; ^※^ VAP: ventilator-associated pneumonia.

**Table 4:** Probiotics and synbiotics in VAP risk: outcomes and limitations.

Study (Year)	Other Important VAP Outcomes	Study Limitations	Reference
Siempos et al. (2010)	1. Exclusion of an RCT ^§^ where probiotics were applied only to the oral cavity. The benefit in reducing VAP ^⁂^ incidence remained (fixed OR ^‡^: 0.62; random OR ^‡^: 0.56). 2. Synbiotic 2000 FORTE subgroup. Analyzing only three RCTs ^§^ using the same regimen showed a more pronounced reduction (OR ^‡^ 0.44 in both models). 3. Excluding a high VAP ^⁂^ incidence RCT ^§^ weakened the effect (fixed OR ^‡^: 0.68; random OR ^‡^: 0.64), losing statistical significance.	1. Study heterogeneity: Variations in patient populations, probiotic dosing, duration, and administration methods may confound results. 2. Data gaps: Limited information on antimicrobial usage and blood culture practices in some trials, affecting safety assessments.	[6]
Bo et al. (2014)	1. Exclusion of a study applying probiotics only to the oral cavity. Significant reduction in VAP ^⁂^ incidence (OR ^‡^ 0.56, 95% CI ^†^ 0.30–1.06). 2. Synbiotic 2000 FORTE trials. Significant VAP reduction (OR ^‡^ 0.44, 95% CI ^†^ 0.24–0.79). 3. Excluding a high VAP ^⁂^ incidence study slightly weakened statistical significance (OR ^‡^ 0.64, 95% CI ^†^ 0.35–1.17). 4. Sensitivity analyses: High-dose probiotics (>10^10^ per dose) showed no significant difference (OR ^‡^ 0.77, 95% CI ^†^ 0.51–1.17). Probiotics given twice daily significantly reduced VAP ^⁂^ (OR ^‡^ 0.64, 95% CI ^†^ 0.43–0.96).	Low evidence quality: Small sample sizes, high methodological heterogeneity, and probiotic strain variations make definitive conclusions difficult.	[30]
Lau et al. (2020)	Only one study mentioned VAP ^⁂^, which was a model-based health economic evaluation (observational study).	1. Very little evidence. 2. High heterogeneity in reporting prevented meta-analysis. 3. Variability in time horizons and payer perspectives affects comparability. 4. Potential funding bias from manufacturer-sponsored studies. 5. Limited data on rare complications, like probiotic-induced infections.	[32]
Su et al. (2020)	Probiotics significantly reduced antibiotic duration for VAP ^⁂^ (mean difference: −1.44 days, 95% CI ^†^ [−2.88, −0.01]).	1. Differences in trial design and VAP ^⁂^ diagnostic criteria may introduce heterogeneity. 2. Subgroup analysis of double-blind studies showed no probiotic effect, suggesting potential overestimation in other studies. 3. Heterogeneous patient populations (trauma, surgery, sepsis) may affect the results. 4. Probiotic strain, dosage, and delivery method variations reduced comparability. 5. Low methodological quality with issues in randomization, allocation concealment, and blinding. 6. Potential publication bias.	[31]
Cheema et al. (2022)	1. Blinding analysis: No significance in VAP ^⁂^ incidence in double-blind studies (RR ^¶^ 0.80, 95% CI ^†^ 0.63–1.01). 2. Type of intervention: Synbiotics were more effective than probiotics (RR ^¶^ 0.50, 95% CI ^†^ 0.32–0.79 vs. RR 0.77, 95% CI 0.63–0.96). 3. Diagnostic criteria: No significance in VAP ^⁂^ incidence between clinical, microbiological, or unspecified criteria. 4. Sensitivity analysis: No significant difference after excluding trials with bias concerns (RR ^¶^ 0.78, 95% CI ^†^ 0.60–1.00).	1. Bias concerns: 66.7% of studies had some bias concerns, with two studies at high risk of bias. 2. Heterogeneity: Significant variability in patient age, probiotic type, dosages, and routes of administration. 3. VAP definition variability: Different diagnostic criteria across studies may affect reported incidence. 4. No studies included immunocompromised patients, limiting applicability. 5. Lack of patient-level data: Meta-analysis was based on aggregate data rather than individual patient data.	[7]
Sharif et al. (2022)	Sensitivity analysis: Excluding high risk-of-bias studies found no significant on VAP ^⁂^ incidence (RR ^¶^ 0.91, 95% CI ^†^ 0.73–1.13).	1. Heterogeneity: Variations in probiotic strains, administration routes, and dosages led to inconsistent VAP ^⁂^ prevention outcomes. 2. Outcome variability: Differences in VAP ^⁂^ definitions across trials affected comparability and reliability. 3. Small sample sizes: Many trials had fewer than 100 patients, increasing type I error risk and limiting statistical power. 4. Patient severity differences: The largest trial (PROSPECT) included sicker patients (mortality 28.1%) compared to other trials (pooled mortality 17.4%), which may contribute to differences in VAP ^⁂^ outcomes.	[33]

^†^ CI: confidence interval; ^‡^ OR: odds ratio; ^§^ RCTs: randomized controlled trials; ^¶^ RR: risk ratio; ^⁂^ VAP: ventilator-associated pneumonia.

## Data Availability

The original contributions presented in the study are included in the article; further inquiries can be directed to the corresponding author.
